# *Houttuynia cordata Thunb*-derived extracellular vesicle-like particles alleviate ischemic brain injury by miR159a targeting ACSL4 to suppress ferroptosis

**DOI:** 10.1186/s13020-025-01193-z

**Published:** 2025-09-01

**Authors:** ShenYang Zhang, ZhiYan Liang, ChunYi Wu, ZiLu Qin, XueWen Wei, YiNing Liu, RuiQi Su, LiLi Li, Bin Sun, LinYan Huang, Wan Wang, JianGang Shen, GuangSheng Wang, SuHua Qi

**Affiliations:** 1 Department of Neurology, The Affiliated Hospital of Xuzhou Medical University, Xuzhou, Jiangsu People’s Republic of China; 2 School of Medicine, Southeast University, Nanjing, Jiangsu People’s Republic of China; 3 School of Medical Technology, Xuzhou Key Laboratory of Laboratory Diagnostics, Xuzhou Medical University, Xuzhou, 221004 Jiangsu People’s Republic of China; 4 Department of Laboratory Medicine, Affiliated Xuzhou Municipal Hospital of Xuzhou Medical University, Xuzhou, Jiangsu People’s Republic of China; 5 School of Chinese Medicine, The University of Hong Kong, Hong Kong SAR, Hong Kong, China; 6 Department of Neurology, The Affiliated Shuyang Hospital of Xuzhou Medical University, Shuyang, 223600 Jiangsu People’s Republic of China

**Keywords:** Cerebral ischemia–reperfusion, HT-EVLP, Ferroptosis, miR159a, ACSL4

## Abstract

**Introduction:**

Neuroprotective agents for acute ischemic stroke often fall short in efficacy due to the blood–brain barrier challenges, lack of target specificity, and limited effectiveness. Recently, plant-derived extracellular vesicle-like particles (EVLP) have shown promise in their multifaceted functions.

**Objectives:**

The neuroprotective advantages that EVLP produced from Houttuynia cordata Thunb against cerebral ischemia/reperfusion injury are investigated.

**Methods:**

The extraction of HT-EVLP was performed using gradient centrifugation and ultracentrifugation, followed by identification of its particle size, morphology, and exosomal marker proteins. Using behavioral tests and a rat model of middle cerebral artery occlusion (MCAO), the neuroprotective attributes of HT-EVLP were assessed. To evaluate the effect of HT-EVLP on ferroptosis and cell survival, the oxygen–glucose deprivation/reoxygenation (OGD/R) induced HT22 cell model was used. Utilizing bioinformatics analysis and small RNA sequencing, the miRNA composition and downstream target genes of HT-EVLP were predicted. The dual-luciferase reporter gene assay was used to confirm that miR159a bound to long-chain acyl-coenzyme A synthase 4 (ACSL4). The impact of miR159a transfection on OGD/R-induced ferroptosis in HT22 cell was also observed.

**Results:**

Using a MCAO model, we found that HT-EVLP preserved blood brain barrier integrity, naturally penetrated the infarct core area, reduced cerebral infarct volume, mitigated neuronal apoptosis and ferroptosis, and facilitated recovery of neuronal function. In vitro studies further revealed that HT-EVLP enhanced cell survival and suppressed ACSL4-mediated ferroptosis in OGD/R-treated HT22 cells. Small RNA sequencing indicated that HT-EVLP are rich in miRNAs, with miR159a, among the top 10, potentially regulating ferroptosis-related pathways and directly binding to the 3’UTR of ACSL4. Overexpression of miR159a reduced Erastin-induced ACSL4 expression and alleviated mitochondrial damage in HT22 cells without causing toxicity.

**Conclusions:**

This study highlights the potential of HT-EVLP as carriers of endogenous miR159a, offering a promising strategy for ischemic brain injury therapy.

## Introduction

Ischemic stroke (IS) is the leading cause of death and disability, and it disproportionately affects low-income countries with inadequate access to healthcare [1, 2]. The challenges of preventing and treating IS are increasing due to the aging population. More than 60% of IS survivors experience varying degrees of functional disabilities within three months post-stroke, significantly affecting their quality of life. Therefore, developing effective neuroprotective agents is crucial for alleviating these disabilities. The main approaches to treating IS include endovascular thrombectomy and intravenous thrombolysis. However, due to time constraints, only about 5% of patients are eligible for these interventions [3]. Furthermore, the blood–brain barrier significantly hinders the effective delivery of most neuroprotective agents to the brain**,** further limiting their therapeutic efficacy and clinical application [4]. Edaravone-Dexborneol is a multi-target neuroprotective medication that has been demonstrated to have good safety and to substantially improve neurological recovery in individuals who suffered an acute IS. Therefore, new neuroprotective agents are essential for reducing the incidence and mortality, improving functional outcomes, and overcoming the limitations of existing therapies for IS.

IS is an extremely complex pathological process involving excitotoxicity, calcium overload, oxidative stress, and inflammation, and ultimately contributes to neuronal death [5, 6]. Apoptosis, necrosis, autophagy, and pyroptosis can lead to neuronal damage independently or synergistically [7]. Ferroptosis, a newly recognized form of programmed cell death dependent on iron ions, is characterized by lipid peroxidation and typically accompanied by the accumulation of reactive oxygen species [8]. In stroke patients, ferroptosis is associated with stroke severity and outcomes, correlating with higher 4-hydroxynonenal and lower soluble transferrin receptor levels [9].This form of cell death primarily affects neurons and has garnered significant attention due to its implications for IS. Moreover, liproxstatin-1 and ferrostatin-1 have effectively alleviated neural damage, significantly reduced cerebral infarcts' size, and improved neurological function [10]. Hence, the therapeutics targeting ferroptosis with good biosafety may provide a promising protective strategy against neuronal damage resulting from cerebral ischemia/reperfusion (I/R) and could emerge as a viable therapeutic approach for IS.

Extracellular vehicles (EVs) are heterogeneous, phospholipid bilayer-enclosed membranous particles with diameters ranging from 30 to 1000 nm [11]. EVs encapsulate proteins, lipids, and nucleic acids (miRNAs, mRNAs, and DNAs), enabling them to transport active biomolecules and treat cerebrocardiovascular disease, tumors, and inflammatory diseases [12–14]. Plant-derived extracellular vesicle-like particles (P-EVLP) have recently gained extensive attention, as they resemble mammalian EVs in size, morphology, and characteristic markers. Preferably, P-EVLP is mainly obtained from edible and medicinal plants, establishing their non-toxic, low-immunogenic, high-yielding, and large-scalability productive characteristics [15]. Moreover, P-EVLP transport bioactive components, including microRNAs, to mammal cells and show their remarkable potential as sustainable, green, and efficient therapeutic drug delivery nanocarriers. For inflammatory response, EVLP from ginger, soybean, blueberry, nuts, and garlic, encapsulated miR159a, aly-miR396a-5p, rlcv-miR-rL1-28-3p, miR5781,miR156e, miR162, miR319d, and peu-miR2916-p3 to modulate NF-κB and NLRP3 signaling pathway [16–23]. We previously isolated and identified EVs from *Momordica charantia*, demonstrating the contribution of miRNA to their anti-tumor [24] and neuroprotective effects [25, 26]. Therefore, P-EVLP is becoming a novel therapeutic alternative to deliver miRNAs.

Houttuynia cordata Thunb (HT) is a perennial herb with rhizomatous characteristics, and primarily native to the mountainous regions of China, Japan, South Korea, and Southeast Asia [27]. HT contains a variety of pharmacological active substances such as volatile oil and flavonoids, according to the Pharmacopoeia of the People's Republic of China, HT has the functions of clearing heat and detoxifying, eliminating carbuncle and draining pus, diuretic and drenching. Recently, it has been reported that HT-derived 2-undecanone potently inhibits lung carcinogenesis through the nuclear factor-erythroid 2 related factor 2-heme oxygenase-1/NAD (P)H:quinone oxidoreductase 1 axis activation [28]. Flavonoid constituents were found to mitigate pneumonia in mice triggered by H1N1 virus, potentially through modulating the biotransformation activities of intestinal microbiota [29]. Given its diverse bioactive components, HT offers a non-toxic therapeutic approach for various ailments and warrants further research. Recently, the neuroprotective role of Houttuynia cordata volatile oil in cerebral ischemia–reperfusion injury in mice has been studied through anti-inflammatory and antioxidant mechanisms [30]. Additionally, Houttuynia cordata is a critical component in several Chinese prescriptions for stroke treatment [31]. Hence, Houttuynia cordata may exert a potential protective effect on stroke. In this study, we tried to characterize HT-EVLP and investigate their therapeutic effects on cerebral I/R injury both in vitro and in vivo, focusing on their capabilities in protecting and penetrating blood brain barrier (BBB), preventing neuronal apoptosis, and inhibiting ferroptosis. Furtherly, the down-regulation of ACSL4 by HT-EVLP derived miR159a, at least partly, contributed to ferroptosis inhibition. These findings aim to provide compelling evidence for the clinical application of HT-EVLP and establish a theoretical foundation for treating IS.

## Materials and methods

### Isolation and characterization of HT-EVLP

Houttuynia cordata Thunb. was harvested fresh from Yunnan, China. The isolation of HT-EVLP involved sequential differential and ultracentrifugation steps [32, 33]. The plant was processed into juice and centrifuged at 1000*g* for 10 min, followed by 3000*g* for 20 min, and subsequently at 10,000*g* for 40 min to separate the supernatant. This supernatant underwent ultracentrifugation at 150,000*g* for 90 min using a Beckman Optima L-100XP centrifuge (Beckman, USA), and was subsequently filtered through a 0.22 μm pore size filter to yield sterile HT-EVLP. The purified HT-EVLP were examined using a transmission electron microscope (Tecnai G 2 Spirit TWIN 120kV, FEI, USA). Particle size distribution was assessed with a Nanoparticle Tracking Analyzer (ZetaView® f-NTA, Particle Metrix, Germany). The presence of exosomal marker CD9, CD63, and TSG101 was confirmed using antibodies from Proteintech (catalog numbers 20597-1-AP, 25,682-1-AP, and 28,283-1-AP, respectively). The concentration of HT-EVLP was quantified using the BCA protein assay kit (P0011, Beyotime).

### Experimental Animals

Sprague–Dawley rats (Male, 230–250 g), sourced from Xuzhou Medical University's Animal Experiment Center, were utilized in this study under protocol 202205A289, sanctioned by the institution’s Animal Ethics Committee. The rats were housed in a regulated environment (24 °C ± 1 °C, 50–60% RH, 06:00–18:00 light cycle), and were randomly assigned to three groups: Sham, MCAO with 2-h occlusion and 72-h reperfusion (M2/R72, referred to as MCAO), and M2/R72 treated with HT-EVLP at 100 μg/kg (referred to as HT-EVLP).

### Middle cerebral artery occlusion (MCAO) model

A 2-h MCAO model followed by 72 h of reperfusion was applied [32]. Rats were initially anesthetized with 4% isoflurane and then maintained at 2%. The left common carotid artery was exposed and temporarily clipped, and a 0.38 mm-tipped silicon-coated suture was advanced from the left external carotid artery into the internal carotid artery to occlude the middle carotid artery origin. Sham animals underwent identical anesthesia and surgical steps without middle carotid artery occlusion. Throughout the surgery, rats were kept on a warming pad with body temperature maintained at 37 °C. Following the occlusion period, the suture was removed to initiate reperfusion.

### Bio-distribution of Dil-HT-EVLP in vivo

The freshly extracted HT-EVLP was suspended in sterile PBS buffer and incubated with 25 μL Dil per 1 mL HT-EVLP at 37 ℃ for 15 min. The mixture was then sonicated and centrifuged; the pellet was resuspended in PBS and stored at – 80 ℃. The Dil-labeled HT-EVLP and Dil dye were injected by tail vein injection, the rats were killed at 24 h after injection, and the organs were captured in three-dimensional optical imaging of small animals (Newton 7.0 FT-100).

### Infarct area measurement

2,3,5-Triphenyltetrazolium Chloride (TTC) Staining was employed to assess cerebral infarct size and location post-ischemia in rats. Following a MCAO/reperfusion period, the animals were euthanized, and their brains rapidly excised and flash-frozen at − 20 °C for 30 min. The frozen brains were sectioned into 2-mm-thick slices and stained with 2% TTC solution (A610558, Shanghai Sangon) at 37 °C for 25 min in darkness. Infarct area percentage was determined using the formula: Infarct area percentage = (uninfarct hemisphere areauninfarct hemisphere area − infarct hemisphere uninfarct area) × 100 [34].

### BBB permeability detection

Evans blue (EB) staining was used to inspect the morphology of BBB disruption. After 72 h of MCAO, rats were anesthetized with pentobarbital (40 mg/kg, ip) and injected with 2% EB solution (3 ml/kg, Beijing Yinuokai Technology Co., Ltd.) via tail vein. After two hours of circulation, rats were perfused with 0.9% normal saline to discard redundant dyes until perfusion fluid was colorless in the right atrium; then, brains were decapitated quickly and divided into consecutive 2 mm-thick coronal slices. The digital photos of coronal slices were recorded.

EB leakage was measured to analyze BBB permeability quantitatively. The brain tissue was weighed and stored at − 80 °C for later analysis. Each hemisphere was mechanically homogenized in PBS at 4 °C, followed by adding an equal volume of 50% trichloroacetic acid solution (Beijing Yinuokai, China). The concentration of EB in the supernatant was determined using a spectrophotometer (Bio-Rad 680, USA) at 620 nm/680 nm wavelengths.

Mice received an intravenous injection of FITC-dextran (30 mg/kg; 46944, Sigma) and were allowed a 20-min circulation time. Euthanasia was performed, followed by cardiac perfusion with 0.9% saline to clear blood from the vasculature. The affected hemisphere of the brain was extracted, weighed, and homogenized. The supernatant was mixed with an equal volume of methanol and centrifuged again at 10,000*g* for 15 min. Fluorescence intensity was quantified using 485 nm/520 nm.

### Brain water content assay

After HT-EVLP treatment, mouse brain tissues were collected for wet weight assessment. Subsequently, tissues were dried at 100 °C to a constant dry weight, with measurements taken to ensure accuracy (error < 0.002 g). The percentage of brain water content was calculated as follows: Brain water content = (wet weight-dry weight)/wet weight × 100% [35].

### Western blotting

The protein was separated and then transferred PVDF membrane (Millipore, MA, USA). After blocking with 5% non-fat milk, the membranes were incubated with primary antibodies overnight at 4 °C. The primary antibodies used were anti-CD9 (Proteintech, 20,597–1-AP), anti-TSG101 (Proteintech, 28,283–1-AP), anti-CD63 (Proteintech, 25,682–1-AP), anti-GPX4 (Proteintech, 67,763–1-Ig), anti-ACSL4 (ABclonal, A20414), anti-TFRC (ABclonal, A5865), and β-actin (Proteintech, 66,009–1-lg) and α-tubulin (Proteintech, 66,031-1-lg) antibodies. Then the membranes were incubated with appropriate secondary antibodies. The blots were visualized using an ECL detection kit (Millipore, KF8001).

### Open field test

The rats were subjected to an open-field test within a 50 cm square arena, enclosed by 50 cm walls, and monitored by a video camera. The arena was sectioned into 16 equal parts, with the inner four sections defined as the central zone. Animals were positioned in the central zone and allowed to roam for a 5-min. We documented their motor activity, encompassing velocity, distance covered, and corner dwelling duration. Each subject underwent a single trial, with the arena being disinfected with 75% ethanol.

### Balance beam test

The assay involved rats traversing a 1-m-long, 1.4-cm-wide beam, with prior training to familiarize them with the task. Scoring criteria ranged from 0 to 6 points: 0 indicated stable balance; 1 indicated clinging to the beam's edge; 2 indicated embracing the beam with one limb dropping; 3 indicated embracing the beam with two limbs dropping or rotating on the beam for over 60 s; 4 indicated efforts to maintain balance but falling off after over 40 s; 5 indicated efforts to maintain balance but falling off after over 20 s; and 6 indicated immediate fall without attempting to balance or cling, lasting under 20 s.

### Laser speckle contrast imaging

Cerebral blood flow was measured by a laser diffusion haemofiltration imager (SIM BFI-HR, XunWei, Wuhan, China). Under pentobarbital (40 mg/kg, ip) anesthesia after 72 h reperfusion, the heads of rats were fixed on a stereotaxic apparatus. The scalp was incised to expose the skull fully after 75% alcohol disinfection. The Doppler probe was affixed to the skull's surface, and the blood flow state was monitored and recorded.

### TUNEL staining

To evaluate neuronal apoptosis, we employed TUNEL staining on rat brain samples. Brains were first fixed in 4% paraformaldehyde and then dehydrated using a 30% sucrose solution before being sectioned into 20 μm slices. The TUNEL procedure was carried out according to the protocol provided with the detection kit (PF00009, Proteintech, USA). Nuclear visualization was achieved with DAPI counterstaining, and samples were examined under a confocal microscope (Stellaris 5, Leica, Germany).

### Immunofluorescence staining

Frozen sections were incubated with 5% blocking buffer for 1 h, added with a primary antibody with appropriate dilution concentration at 4 ℃. Then, the secondary antibodies were incubated for 1 h. The anti-quencher containing DAPI was incubated for another 15 min, and observed under a fluorescence microscope.

### Establishment of in vitro ischemia–reperfusion model

The HT22 hippocampal neuronal cell line was maintained in DMEM medium (Keygen KGM12800-500, China) containing 10% FBS. An oxygen–glucose deprivation/reoxygenation (OGD/R) model was established. Briefly, cultured cells were washed twice with PBS and transferred to glucose-free DMEM medium. The cells were then placed in a hypoxia chamber (ESCO, Singapore) with a controlled gas mixture of 1.2% O_2_ and 5% CO_2_ for 9 h. After the hypoxic exposure, cells were reoxygenated by replacing the medium with complete DMEM and incubating under normoxic conditions for 24 h. The experimental design comprised three groups: (1) control group (normoxic conditions), (2) OGD/R group, and (3) OGD/R group pretreated and post-treated with 10 μg/ml HT-EVLP.

### Calcein AM/propidium iodide (PI) staining

HT22 hippocampal neurons were cultured in laser confocal dishes and subjected to various experimental treatments. Post-treatment, cells were stained with PI and calcein AM solutions (Dojindo, Japan) for a 15-min incubation. Here, PI’s red fluorescence indicated cell death, while calcein AM’s green fluorescence signified cell viability. Fluorescence microscopy was employed to document the cellular responses.

### Measurement of lipid peroxidation

HT22 cells were cultured in laser confocal dishes and subjected to OGD/R and HT-EVLP treatment. Subsequently, the cells were stained with 10 μM C11-BODIPY 581/591 fluorescent probe (Thermofisher, USA) for 20 min at 37 ℃ in the dark. Cellular fluorescence was visualized and recorded using a Leica STELLARIS 5 confocal microscope (Germany).

### Malondialdehyde (MDA), glutathione (GSH), and superoxide dismutase (SOD) determination

MDA, GSH, and SOD levels were measured to evaluate lipid peroxidation levels in brain tissue and neurons. The MDA kit (MM-0385R2) and GSH kit (ADS-F-G001) were obtained from Meimian (Shanghai, China), while the SOD kit (A003-1, A001-3) was sourced from Jiancheng (Nanjing, China).

### miRNA sequencing and miRNA target gene analysis

The miRNA sequencing of HT-EVLP was performed by Beijing Genomics Institute (Shenzhen, China). The miRNA target gene analysis was carried out using the miRanda and TargetScan web tools. The intersection of miRanda target genes and TargetScan were the miRNA target gene prediction results.

### miR159a transfection

miR159a was synthesized by Sangon (Shanghai, China). HT22 cells were incubated and transfected with miR159a using Lipofectamine 2000 (Invitrogen, USA) for transfection. After 48 h of transfection, the cells were subjected to OGD/R.

### Dual-Luciferase reporter gene assay

The wild-type (WT) and 3’-UTR fragment mutants of ACSL4 at the miR159a binding site synthesized by Genechem (Shanghai, China) were cloned in SV40-firefly_Luciferase-MCS. The HEK293T cells were transfected with the miR159a mimic, and GV 272-ACSL 4-WT (5’ATTCTTCATGAAAATCCAAA3’) or GV 272-ACSL 4-Mut (5’ ATTCTTCATGAAACGAACCA 3’) reporter plasmid and the Renilla_Luciferase plasmid. Luciferase activity was measured using a GloMax 20 / 20 luminometer Dual-Glo ™ Luciferase Assystem (E2920, Promega).

### Real-time PCR

Specific miRNA was converted to cDNA using the industrial miRNA first strand cDNA synthesis (stem-loop method) kit (miRNA5813b RT primer: GTCGTATCCAGTGCAGGGTCCGAGGTATTCGCACTGGATACGACTCCATG). The expression of miR159a was measured using the High Sensitivity PCR kit (Novazyme, Nanjing, China). The miR159a Forward primer: UUUGGAUUGAAGGGAGCUCUA; Reverse primer: AAACCUAACUUCCCUCGAGAU.

### LDH assay

Lactate dehydrogenase (LDH) activity was determined utilizing the Pierce LDH Cytotoxicity Assay Kit from ThermoFisher Scientific, adhering to the provided guidelines. Collected samples comprised 50 µl of conditioned medium and 50 µl of cell lysate, obtained after lysing the cells with 10 µl of the kit's lysate buffer at 37 °C for 45 min. The LDH assay was conducted on both conditioned medium and lysate, with absorbance readings taken at 490 and 680 nm using a SpectraMax iD5 plate reader (Molecular Devices, San Jose, CA).

### CCK-8 assay

HT22 cells were plated in 96-well plates and subjected to various treatments. They were then treated with 10 μL of CCK-8 reagent (MCE, NJ, USA) and incubated for 4 h. Absorbance readings were taken at 450 nm with a microplate spectrophotometer.

### Mitochondrion morphorlogy detection

HT22 cell pellets were initially fixed in 3% glutaraldehyde at 4 °C for 24 hours, followed by postfixation in 2% osmium tetroxide at 4 °C for an additional 24 hours. Dehydration was carried out using ethanol series with propylene oxide, and the pellets were then embedded in Epon 812 resin (Merck, USA) and incubated at 60 °C for 48 hours. Ultrathin sections, 80 nm thick, were mounted on 200-mesh copper grids and examined using an HT7700 TEM (Hitachi, Tokyo, Japan). Image J software (NIH, USA) quantified mitochondrial cristae density and swelling.

### Data analysis

Data were presented as mean ± standard deviation. Statistical analysis was carried out using GraphPad Prism 9.0 software (GraphPad Software Incorporation, California, United States). The data analysis was performed by one-way or two-way ANOVA, followed by Dunnett's t post-hoc test or the Newman-Keuls test. *P* < 0.05 was considered a significant difference.

## Results

### Isolation and characterization of HT-EVLP

**Fig. 1:**
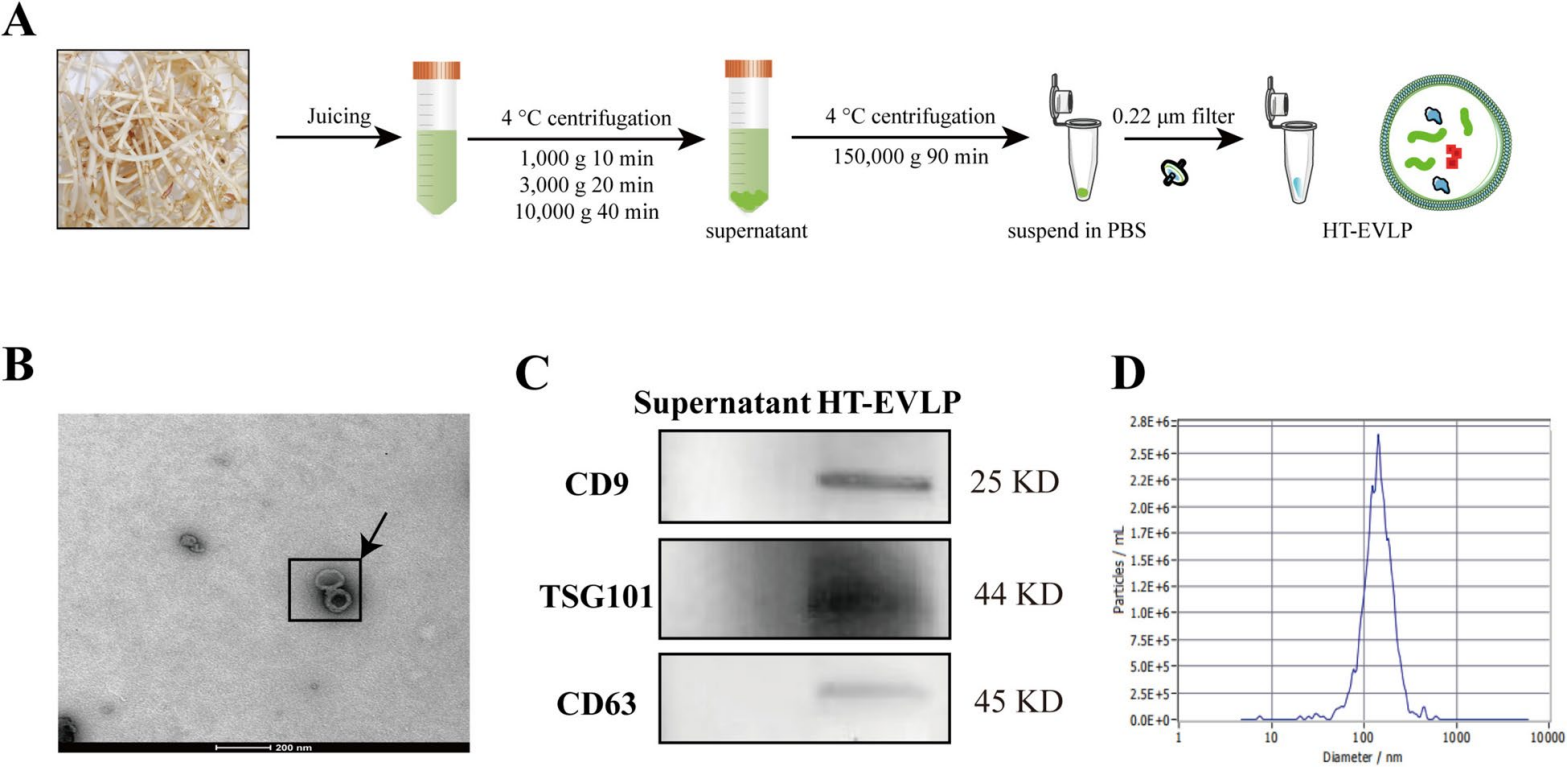
Isolation and characterization of HT-EVLP. **A** Schematic representation of HT-EVLP isolation from *Houttuynia cordata Thunb* (HT) juice. **B** HT-EVLP were photographed by transmission electron microscopy (TEM). Scale bar = 200 nm. **C** The expression of EVs marker proteins CD9, CD63 and TSG101 in HT-EVLP was detected using western blotting, the supernatant of isolated HT-EVLP was used as a negative control. **D** Nanoparticle tracking analysis (NTA) showed the avarege size of HT-EVLP was around 143 nm

Here, HT-EVLP were isolated from fresh *Houttuynia cordata Thunb* using low-speed gradient centrifugation combined with ultracentrifugation. The sterilized HT-EVLP was obtained by passing through a 0.22 μm filter (Fig. 1A). HT-EVLP showed the lipid bilayers and the typical cup-like morphology (Fig. 1B). Like mammal EVs, HT-EVLP also expressed EV marker proteins, including CD9, TSG101, and CD63 (Fig. 1C). HT-EVLP ranged in size from 100 to 200 nm, with an average particle diameter of 143.8 nm (Fig. 1D). These findings demonstrate that HT-EVLP, exhibited the characteristic features of EVs.

### HT-EVLP improved locomotion activity and reduced infarct size in I/R rats.

**Fig. 2:**
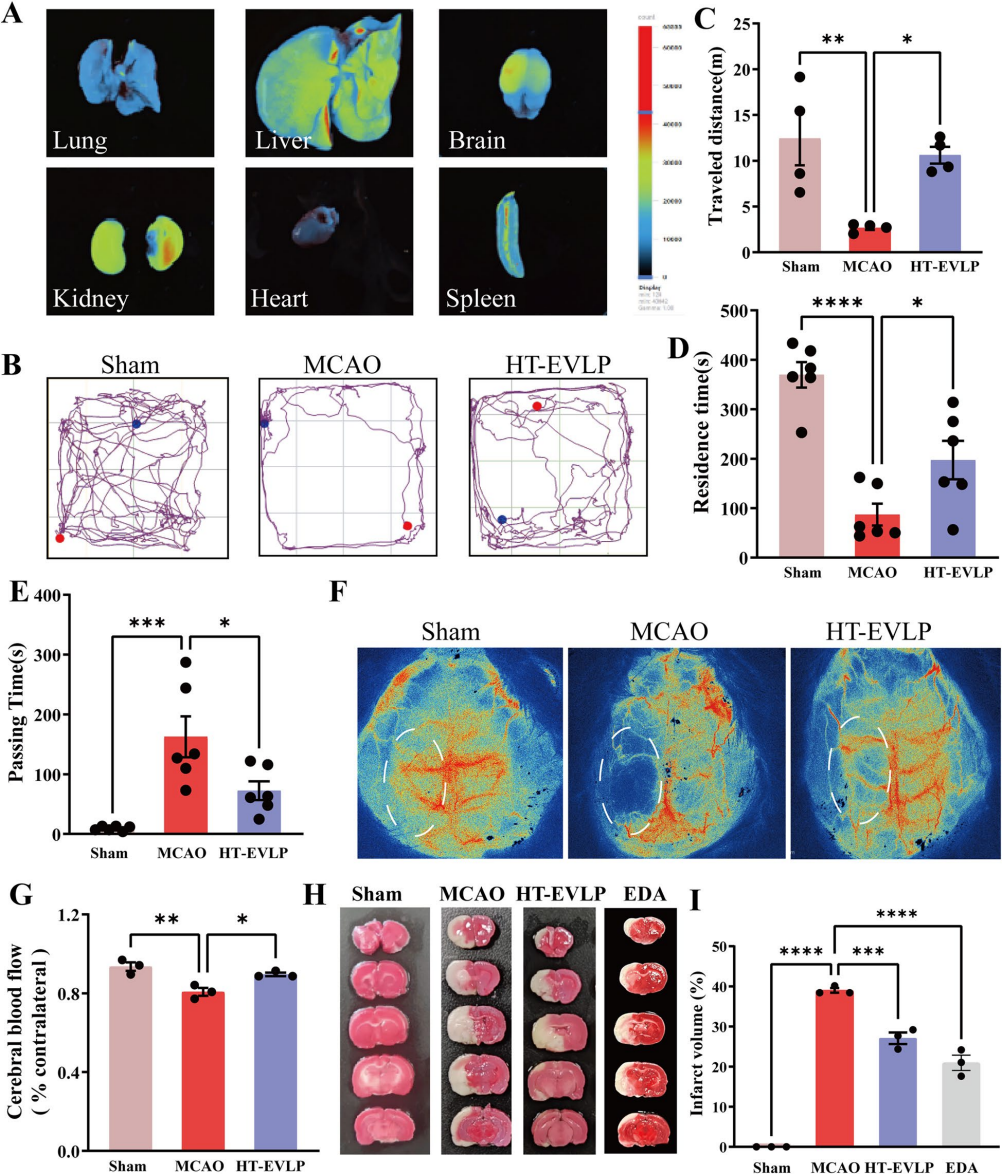
HT-EVLP improves locomotion activity and reduces infarct size in I/R rats. **A** In vivo distribution of HT-EVLP. The MCAO rats was injected with Dil-HT-EVLP, after 24 h, the brain, heart, liver, spleen, lung, and kidney was obtained and imaged with an Vilber Bio Imaging system. **B** The representative images of the movement trajectory plot of rats in open field tests. **C** Statistics of the total distance in the open field test traveled by the rats from Sham (n = 4), MCAO (n = 4) and HT-EVLP (n = 4). **D** Statistics of the residence time on the rotarod by the rats from Sham (n = 6), MCAO (n = 6) and HT-EVLP (n = 6). **E** Statistics of the time to pass the balance beam by the rats from Sham (n = 6), MCAO (n = 6) and HT-EVLP (n = 6). **F, G** Distribution and statistics of cerebral blood flow in rats from three groups (n = 3). **H** Representative images of TTC staining or rat brains from the MCAO rats treated with HT-EVLP and Edaravone. Edaravone was used as a positive drug. **I** The statistical analysis of the TTC staining (n = 3). *P < 0.05, **P < 0.01, ***P < 0.001, ****P < 0.0001 *vs* MCAO

To investigate the natural targeting property of HT-EVLP to the ischemic cortex, we tested the biodistribution of HT-EVLP in MCAO rats. By intravenous injection with Dil-labeled HT-EVLP after 2 h of ischemia, the brain, heart, liver, spleen, lung, and kidney were harvested after 24 h of reperfusion. Using an in vivo imaging system, we found that Dil-labeled HT-EVLP can cross the BBB and primarily accumulate in the infarct area on the left side of the brain (Fig. 2A). The systemic injection of Dil-labeled HT-EVLP also caused their abundance in the liver and kidney, where they are probably metabolized.

The intrinsic targeting of the infarct area of HT-EVLP guaranteed their neuroprotective potential. Indeed, HT-EVLP treatment for 72 h significantly improved the locomotor activity of MCAO rats in the open-field test, evidenced by the increase in total distance moved (Fig. 2B, C). HT-EVLP treatment extended the rotarod’s residence time and reduced the balance beam's passing time (Fig. 2D, E). These behavioral tests indicated that HT-EVLP treatment enhanced the motor coordination ability of rats with cerebral I/R injury and promoted their neurological function recovery. Furtherly, the cerebral blood flow and cerebral infarct area were measured to evaluate the effect of HT-EVLP on the blood flow supply in the ipsilateral cortex. HT-EVLP could restore the cerebral blood flow (Fig. 2F, G). Also we tested the effect of 100 μg/kg HT-EVLP on infarct area in MCAO rats, and 3 mg/kg Edaravone was used as a positive control. 100 μg/kg HT-EVLP reduced the infarct area significantly, although 3 mg/kg edaravone worked better on mitigating cerebral infarction (Fig. 2H, I). Overall, HT-EVLP can alleviate cerebral injury and promote neurological function recovery caused by I/R.

### HT-EVLP protected against BBB and neuronal damage after cerebral I/R in rats

**Fig. 3:**
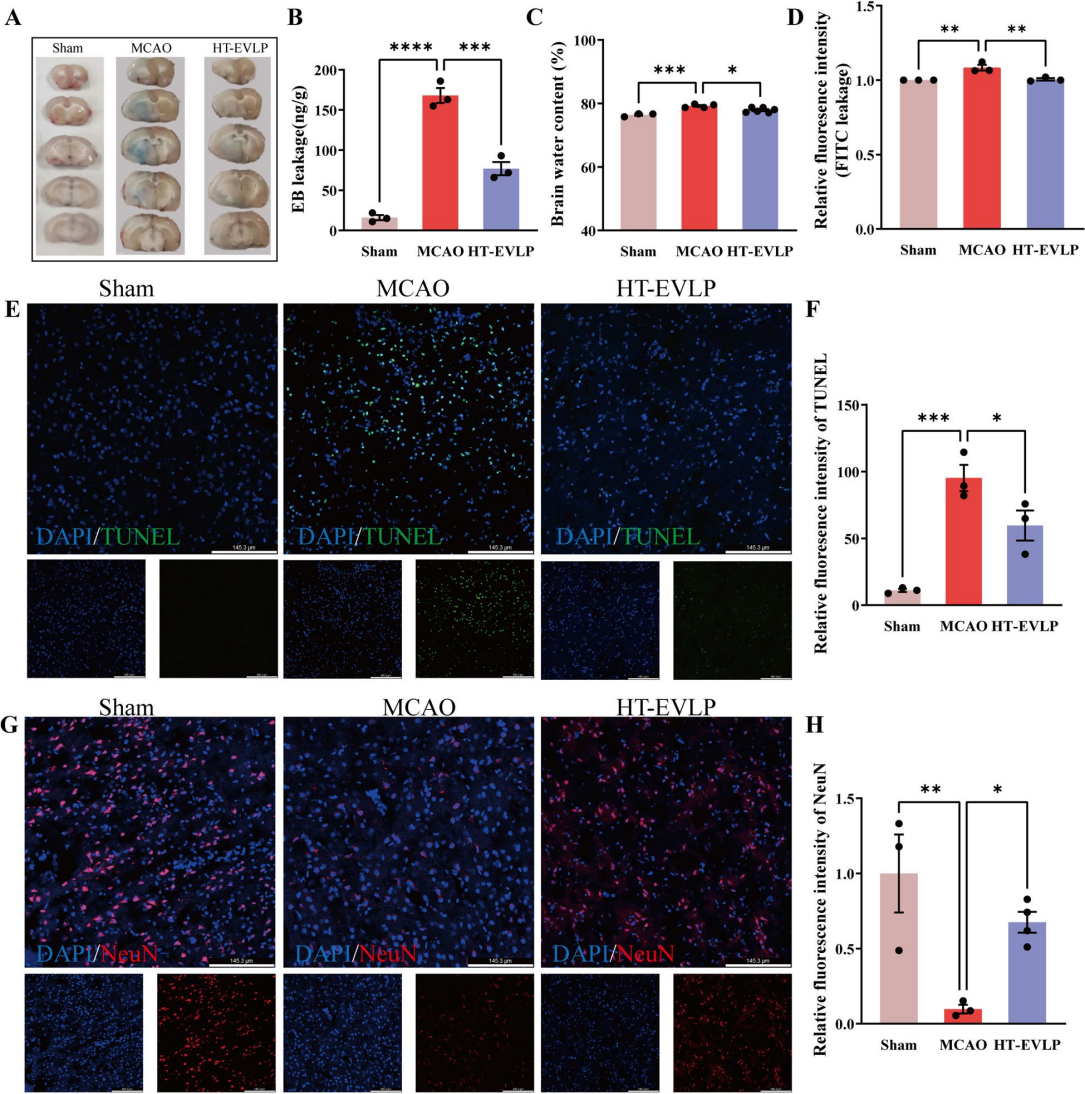
HT-EVLP protect against BBB and neuronal damage in MCAO rats. **A, B** The representative images and statistical analysis of rat brain EB leakage (n = 3). **C** Statistical analysis of brain water content in rats from Sham (n = 3), MCAO (n = 4) and HT-EVLP (n = 6). **D** Statistical analysis of FITC leakage in rat brain (n = 3). **E, F** Immunofluorescence and statistical analysis of TUNEL positive cells in the frozen sections of ischemic brains. **G, H** Immunofluorescence and statistical analysis of NeuN positive cells in the frozen sections of ischemic brains. *P < 0.05, **P < 0.01, ***P < 0.001, ****P < 0.0001 *vs* MCAO

We performed an EB leakage assay to evaluate BBB permeability. No EB leakage was observed in the brains of Sham rats, indicating that the BBB was intact and undamaged. A significant increase in EB leakage could be observed on the ischemic side of the brains of rats in the MCAO group, indicating that the integrity of the BBB had been damaged. A significant decrease in EB leakage was detected on the ischemic side of the brains of rats injected with HT-EVLP, and the EB quantification of the ischemic brain was consistent with previous results (Fig. 3A, B). After ischemic stroke, the destruction of the extracellular matrix and increased vascular permeability lead to brain edema, with a significant increase in brain water content. HT-EVLP treatment significantly reduced brain water content in MCAO rats (Fig. 3C). Then, the FITC-dextran leakage assay was furtherly verified that HT-EVLP treatment abrogated the increase of the fluorescence intensity of FITC in the brain of MCAO rats (Fig. 3D). All these results suggest that HT-EVLP treatment effectively reduces BBB damage following I/R injury. Growing evidence indicates that neuronal apoptosis is the main form of I/R injury, particularly in ischemic regions, where it leads to increased neuronal death and exacerbates neurological dysfunction [36, 37]. Herein, inhibition of neuronal apoptosis and the number of survival neurons represented the neuroprotective effect of HT-EVLP. Unexpectedly, we found that TUNEL staining positive cells in the ischemic cortex were strikingly decreased in HT-EVLP-treated MCAO rats (Fig. 3E, F). Consistently, the number of NeuN-positive (alive) neurons was notably decreased in MCAO rats, while HT-EVLP treatment significantly increased the number of surviving neurons (Fig. 3G, H). These results suggest that HT-EVLP effectively protects neurons from apoptosis and promotes neuronal survival following MCAO, underscoring their potential neuroprotective effects.

### HT-EVLP prevented ferroptosis caused by cerebral I/R injury.

**Fig. 4:**
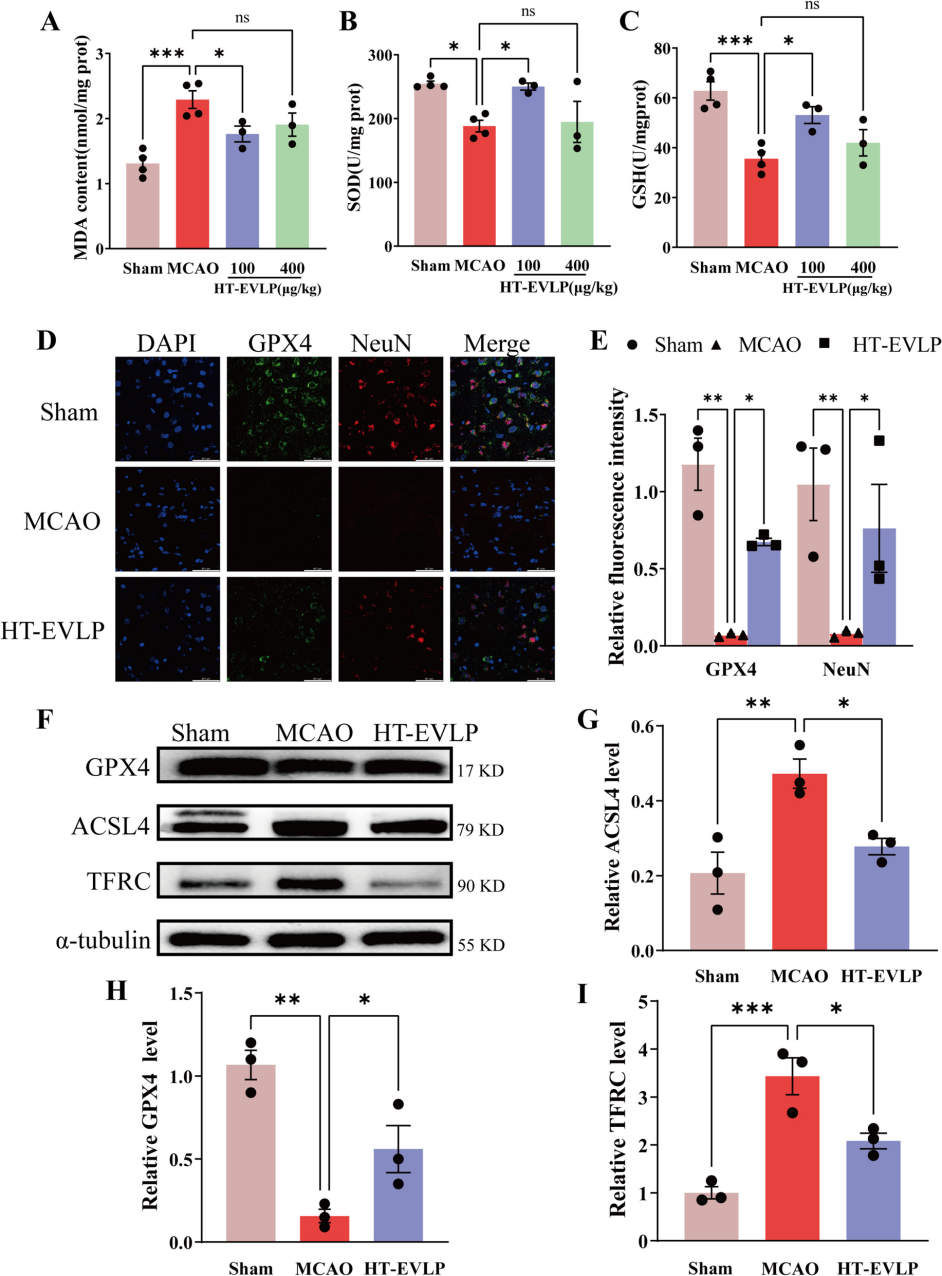
HT-EVLP reduce ferroptosis levels after cerebral ischaemia–reperfusion in rats. **A–C** The level of total MDA(A), SOD(B) and GSH(C) in the cortex from Sham (n = 4), MCAO (n = 4), 100 μg/kg HT-EVLP (n = 3) and 400 μg/kg HT-EVLP (n = 3) groups. **D, E** Immunofluorescence and statistical analysis of GPX4^+^NeuN^+^ cells in the frozen sections of ischemic brains. **F–I** Effect of HT-EVLP on GPX4, ACSL4 and TFRC expression in the ischemic cortex determined by western blotting analysis. *P < 0.05, **P <0.01, ***P < 0.001 *vs* MCAO

Oxidative stress is particularly relevant in ferroptosis, driven by iron-dependent lipid ROS accumulation, which is implicated in stroke [38, 39]. The oxidative stress changes of cerebral I/R injury in rats were evaluated by detecting the contents of GSH, SOD, and MDA (a decomposition product of lipid peroxides) in the cerebral cortex of rats. The results showed that GSH and SOD were significantly decreased. MDA was increased significantly after cerebral I/R, while HT-EVLP treatment mitigated the levels of decreased GSH, SOD, and the elevated MDA induced by cerebral I/R in rat brain cortex (Fig. 4A–C). The above results suggest that HT-EVLP can reduce cerebral I/R injury in rats by decreasing oxidative stress. Immunofluorescence staining of frozen rat brain sections revealed a significant reduction in GPX4⁺/NeuN⁺ cells following MCAO. However, intravenous injection of HT-EVLP resulted in a marked increase in GPX4⁺/NeuN⁺ cells (Fig. 4D, E). In the rat hippocampus, the GPX4 protein level significantly decreased after MCAO compared to the Sham group, while the levels of ACSL4 and TFRC proteins were substantially elevated. These findings indicate an increased occurrence of ferroptosis following cerebral I/R injury. However, treatment with HT-EVLP effectively reversed these changes by increasing GPX4 levels and reducing ACSL4 and TFRC levels (Fig. 4F–I). In summary, these results demonstrate that HT-EVLP exerts a protective effect against cerebral I/R injury by inhibiting ferroptosis and reducing oxidative stress.

### HT-EVLP protected against OGD/R-induced ferroptosis in HT22 cells

**Fig. 5:**
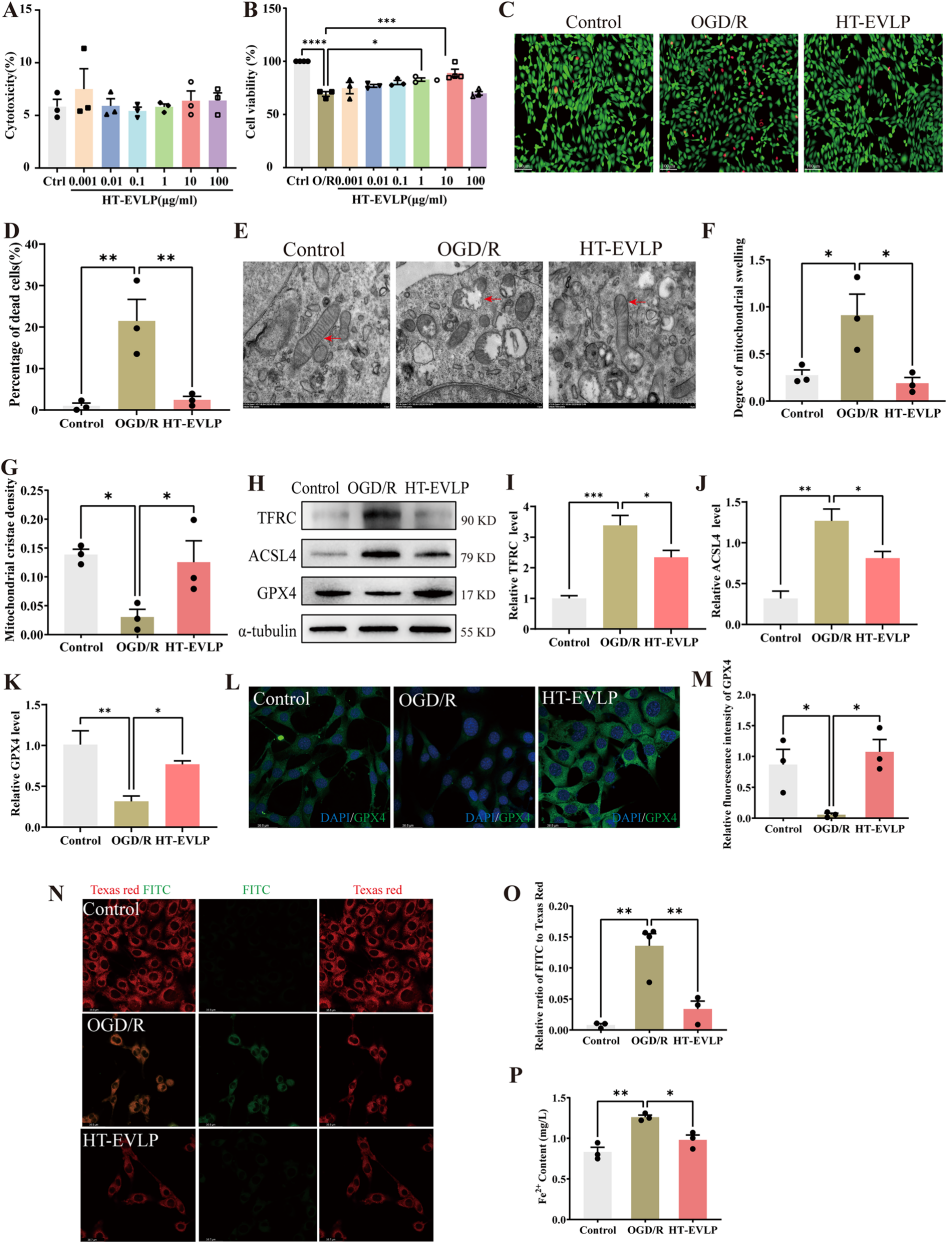
HT-EVLP inhibit OGD/R-induced ferroptosis in HT22 cells. **A** Statistical analysis of cellular LDH release in HT22 cells treated with HT-EVLP in different concentrations. **B** Statistical analysis of the effect of HT-EVLP on the cell viability in OGD/R-treated HT22 cells. **C, D** Calcein-AM/PI staining showed the live (green) and dead (red) cells and statistical analysis. *P < 0.05, **P < 0.01, ***P < 0.001, ****P < 0.0001 *vs* OGD/R (n = 3). **E–G** Mitochondrial morphorlogy detection by TEM (**E**) and statistical analysis of mitochondrial swelling (**F**) and mitochondrial cristae density (**G**). **H**–**K** Effect of HT-EVLP on GPX4, ACSL4 and TFRC expression in OGD/R-treated HT22 cells. **L**, **M** Immunofluorescence staining and statistical analysis of the effect of HT-EVLP on the GPX4 expression in OGD/R-treated HT22 cells. **N, O** BODIPY 581/591C11 staining and statistical analysis of lipid peroxidation (the intensity of FITC to Texas red) in HT22 cells. FITC: oxidized lipids, Texas red: non-oxidized lipids. **P** Statistical analysis of the effect of HT-EVLP on the intracelluar ferrous concentration in OGD/R-treated HT22 cells. *P < 0.05, **P < 0.01 *vs* OGD/R (n = 3)

To determine whether HT-EVLP has any cytotoxicity on normal neuronal cells, we determined the lactate dehydrogenase (LDH) release in HT22 cells treated with HT-EVLP in a serial of concentration (0.001, 0.01, 0.1, 1,10,100 μg/ml). The results indicated that HT-EVLP, at all tested concentrations, did not significantly induce neuronal cell death (Fig. 5A). CCK8 assay was used to determine the optimal therapeutic concentration of HT-EVLP in OGD/R-treated HT22 cells. The results showed that 1 and 10 μg/ml HT-EVLP significantly increased HT22 cell activity, especially 10 μg/ml (Fig. 5B). Next, it was further verified with Calcein/PI cell activity and cytotoxicity assay. The results showed that the PI fluorescence was significantly enhanced by OGD/R treatment, which could be reversed by HT-EVLP treatment, indicating that HT-EVLP could reduce OG/R-induced HT22 cell death (Fig. 5C, D). These results collectively demonstrate that 10 μg/ml HT-EVLP could enhance the survival of HT22 cells after OGD/R without causing cytotoxicity to normal HT22 cells.

Using TEM, we observed the ultrastructure of HT22 cells after OGD/R. The degree of mitochondrial swelling and cristae density was gained by measuring the ratio of the long diameter to the short diameter of mitochondria and the ratio of the area of mitochondrial cristae to the overall area of mitochondria. As shown in Fig. 5E–G, OGD/R caused the mitochondrial morphology of HT22 cells damage, including the rupturing of the outer membrane and the disappearance or reduction of mitochondrial cristae. Treatment with HT-EVLP reduced the degree of mitochondrial swelling, restored mitochondrial cristae density, and mitigated substantial mitochondrial injury induced by OGD/R. These findings indicate that HT-EVLP protects HT22 cells against OGD/R by alleviating mitochondrial damage, a critical factor in the process of ferroptosis.

Then, the level of GPX4, ACSL4, and TFRC was detected. The analysis revealed that in HT22 cells subjected to OGD/R, the levels of the ferroptosis-related protein GPX4 were significantly reduced, while ACSL4 and TFRC levels were markedly increased. Treatment with HT-EVLP restored GPX4 expression and reduced the levels of ACSL4 and TFRC (Fig. 5H–K). Additionally, the fluorescence intensity of GPX4 in OGD/R treated HT22 cells was also reversed by HT-EVLP (Fig. 5L, M).The intracellular lipid peroxidation was also examined using BODIPY 581/591 Cl1 staining. After OGD/R, the non-oxidized lipids (red fluorescence) were decreased, and the oxidized lipids (green fluorescence) were increased, indicating that lipid peroxidation was significantly increased. HT-EVLP treatment decreased oxidized lipids (green fluorescence), suggesting reduced lipid peroxidation (Fig. 5N, O). Besides, intracellular divalent iron levels, which increased significantly after OGD/R, were attenuated by HT-EVLP treatment (Fig. 5P). These findings indicate that ferroptosis is activated in HT22 cells following OGD/R, and HT-EVLP effectively reverses ferroptosis.

### HT-EVLP-derived miR159a specifically degrade ACSL4 to inhibit ferroptosis

**Fig. 6:**
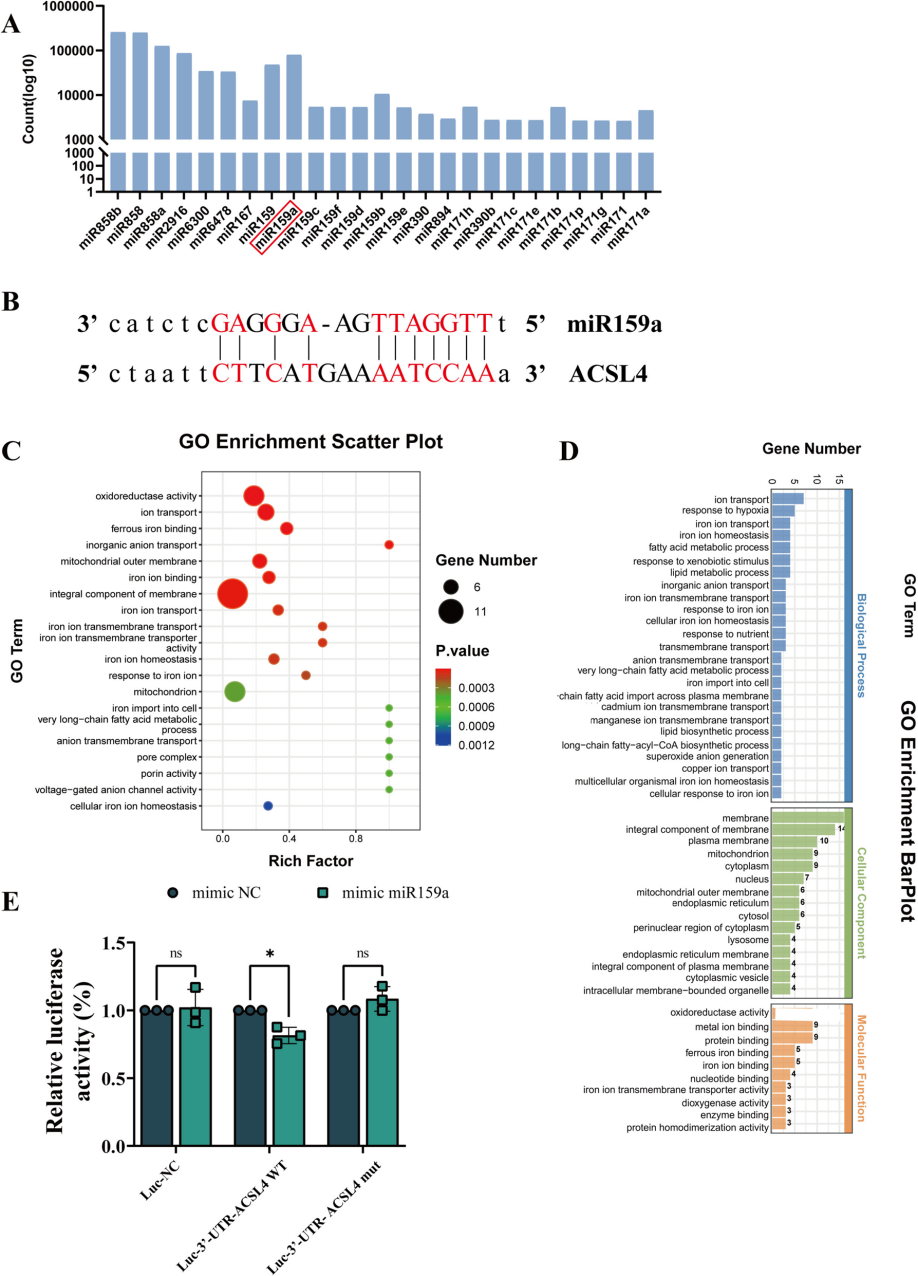
Small RNA sequences of HT-EVLP indicate that ACSL4 is a direct target gene of miR159a. **A** Compositional analysis of small RNAs from HT-EVLP. **B** Prediction of miR159a and ACSL4 3’UTR sequences by bioinformatics analysis. **C, D** GO analysis on the clustered metabolic pathways of the miR159a-targeted downstream genes. **E** Dual luciferase assay validates the bound of miR159a to ACSL4 3’UTR

To precisely elucidate the neuroprotective mechanisms of HT-EVLP in I/R-induced ferroptosis, we conducted a compositional analysis of HT-EVLP at UW Genetics, identifying 25 miRNAs present in high abundance (Fig. 6A).

Using bioinformatics tools to explore potential biological functions, we predicted the interactions between HT-EVLP-derived miRNAs and their target genes. The TargetScan and Mirnada databases were employed to analyze the complementary pairing between plant miRNAs and mammalian target genes, revealing that miR159a could bind to the 3' untranslated region (UTR) of ACSL4 (Fig. 6B). GO enrichment analysis indicated that miR159a is involved in several ferroptosis-related processes, including oxidoreductase activity, ferrous iron binding, mitochondrial outer membrane, iron ion transmembrane transport, and ion homeostasis (Fig. 6C, D). To validate whether miR159a binds to the 3’ UTR of ACSL4, we performed a dual luciferase (firefly luciferase and Renilla luciferase) reporter assay, which confirmed that miR159a significantly reduced the firefly luciferase fluorescence activity in ACSL4 wild-type (WT) plasmid transfected cells. At the same time, no significant effect was observed on the ACSL4 mutant (Mut) plasmid-transfected cells. This result confirmed that miR159a inhibits ACSL4 expression by binding to its 3'-UTR (Fig. 6B, E).

### miR159a inhibited Erastin-induced ferroptosis by reducing ACSL4 expression level

**Fig. 7:**
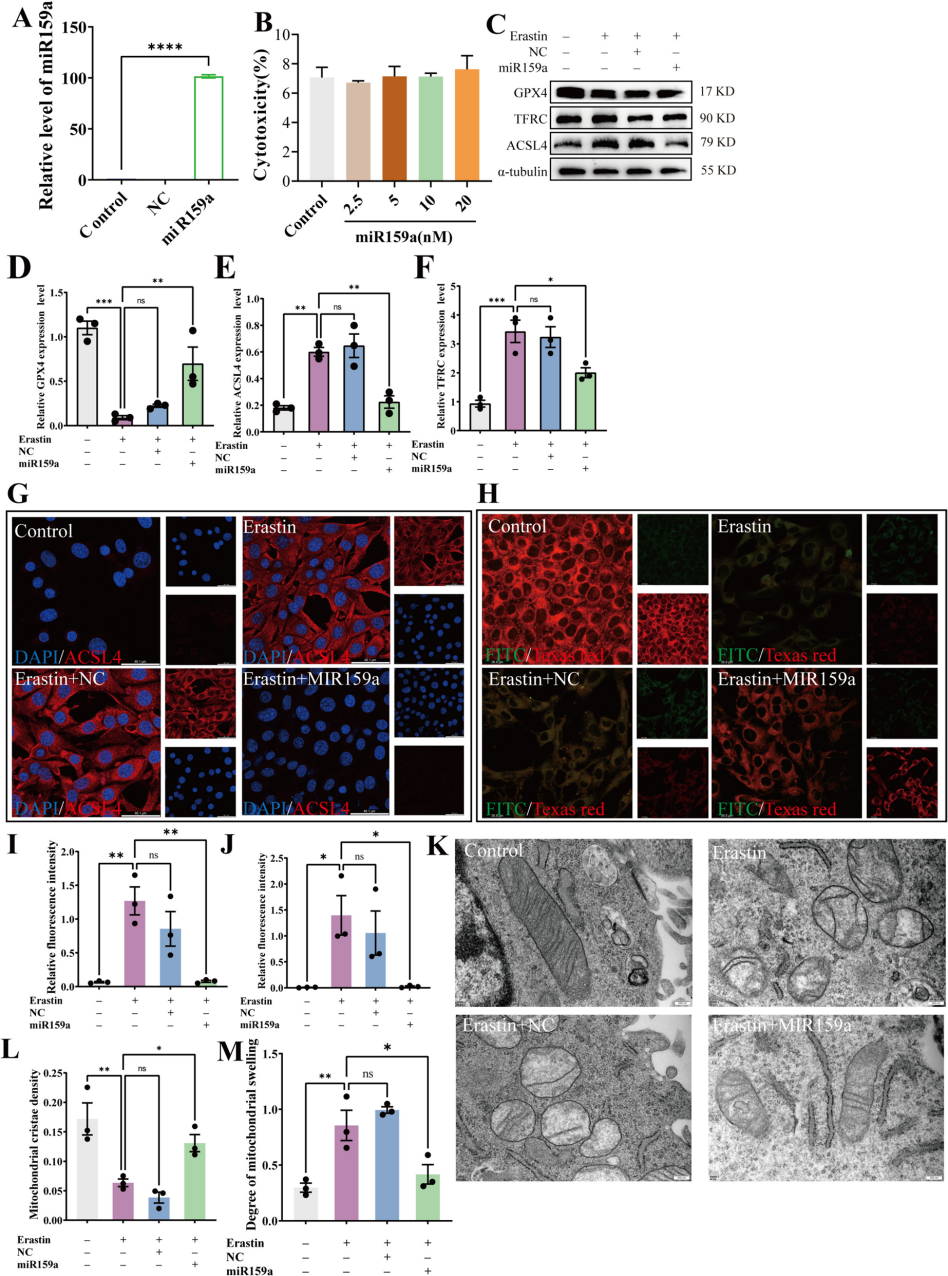
miR159a inhibits Erastin-induced ferroptosis in neuronal HT22 cells. **A** PCR validation of miR159a transfection into HT22 cells. **B** Statistical analysis of the cytotoxicity of miR159a on HT22 cells. **C–F** Effect of HT-EVLP on GPX4, ACSL4 and TFRC expression in Erastin-induced HT22 cells. **G, I** Immunofluorescence and statistical analysis of ACSL4 in Erastin-induced HT22 cells treated with HT-EVLP. **H, J** Immunofluorescence staining and statistical analysis of lipid peroxidation in Erastin-induced HT22 cells treated with HT-EVLP. **K–M** Mitochondrial electron transmission microscopy and statistical analysis mitochondrial cristae density (**L**), mitochondrial swelling (**M**). *P < 0.05, **P < 0.01, ***P < 0.001 *vs* OGD/R (n = 3). NC indicates miRNA mimic negative control

Next, we successfully transfected miR159a into HT22 cells verified via RT-qPCR (Fig. 7A). To explore whether HT-EVLP-derived miR159a has cytotoxicity on normal neuronal cells, we determined the LDH release from HT22 cells. The results showed that overexpression of all concentrations (2.5, 5, 10, 20 nM) of miR159a did not significantly induce LDH release in HT22 cells, suggesting miR159a has no cytotoxicity on normal neuronal cells (Fig. 7B). Western blotting detection and immunofluorescence staining were used to observe the effect of miR159a on ACSL4. The expression level of ACSL4 was significantly suppressed after transfection with 5nM miR159a (Fig. 7C, E). The levels of the other ferroptosis-related proteins were also detected. GPX4 protein levels were decreased, and TFRC protein levels were increased after Erastin stimulation, which could be reversed by miR159a transfection. In contrast, no significant changes were produced in the nonsense sequence (NC) group (Fig. 7C–F). Immunofluorescence staining further confirmed the anti-ferroptosis effect of miR159a, as Erastin stimulation led to a significant decrease in ACSL4 (indicated by red fluorescence) and an increase in lipid peroxidation (indicated by increased green fluorescence), which was reversed by miR159a transfection (Fig. 7G–J). Erastin also induced significant mitochondrial damage, characterized by the loss of mitochondrial cristae and noticeable swelling. However, transfection with miR159a mitigated these mitochondrial defects, restoring cristae density and reducing mitochondrial swelling (Fig. 7K–M). These findings suggest that miR159a can inhibit Erastin-induced ferroptosis in neuronal HT22 cells, thereby reducing neuronal damage by suppressing ACSL4 expression.

## Discussions

To date, developing novel neuroprotective agents has encountered significant obstacles. After decades of investigation, P-EVLP has emerged as a promising candidate for treating ischemic brain injury, outperforming conventional natural compounds. In this study, we purified the EVLP from a traditional medicinal plant, H. cordata. Excitedly, intravenous administration of HT-EVLP mitigates ischemia-induced neurological function deficit in a rat ischemic brain injury model. Their neuroprotection could be explained from three dimensions. First, HT-EVLP treatment quickly penetrates the BBB and protects BBB integrity. Second, HT-EVLP treatment reduces neuronal apoptosis and ferroptosis. Third, miR159a derived from HT-EVLP targets and degrades ACSL4, at least partly contributing to neuronal ferroptosis inhibition.

During ischemic stroke, the integrity of the BBB is compromised, leading to dysfunction and exacerbating the disease. Its structure and biochemical characteristics ensure a homeostatic environment for normal neuronal function by controlling the precise exchange of substances and energy between the blood and the brain. However, these unique properties also limit the penetration of approximately 98% of therapeutic agents for brain lesions [40]. H.cordata is an edible medicinal plant in China and India known for its diverse biochemical constituents, including alkaloids, essential oils, and flavonoids associated with its medicinal properties [41]. Abundant evidence shows that the whole plant of H. cordata or its extract has notable pharmacological effects. However, EVs derived from HT still need to be better understood. The small size of P-EVLP enhances their ability to freely penetrate tissue barriers, including BBB, skin, and intestinal barriers [42]. The HT-EVLP in this study are classified as small EVs with a size of less than 200 nm. This characteristic lays the groundwork for HT-EVLP quickly crossing BBB.

P-EVLP is also well recognized for naturally targeting specific injury sites, although the underlying mechanism remains unclear. For example, *Morinda Officinalis* and *Rhizoma Drynariae*-derived nanovesicles were bone-targeted to reverse osteoporosis [43]. Tea leaves-derived nanotherapeutics were gut-targeted to cure colitis and colon tumor [44, 45]. With its natural biological activity and multi-target protective properties, P-EVLP has shown significant potential in the prevention and treatment of cerebral ischemia–reperfusion injury. Its effects cover multiple levels, such as oxidative stress inhibition, inflammation regulation, BBB protection, and neurogenesis. Although exosomes from foam cells, mesenchymal stem cells, neural stem cells, and microglia have shown neuroprotection in ischemic stroke, P-EVLP is seldom reported, excepted Panax notoginseng and Momordica charantia [32, 46–50]. Our previous findings supported that EVLP from *Momordica charantia* mainly accumulated in the ischemic brain and glioma to exert neuroprotection and anti-glioma effects [24, 32]. Although engineered P-EVLP showed enhanced target ability, for example, modifying the surface of red cabbage-derived EVs with hyaluronic acid, facilitating their binding with CD44 in colon epithelial cells [51]. However, compared with traditional chemo-synthetic nanoparticles or liposomes, the natural targeting of P-EVLP was more acceptable without potential toxicity during engineering.

Although anti-apoptosis is one of the important mechanisms of neuroprotection, in recent years, the role of ferroptosis regulation in treating stroke has also attracted increasing attention. Key genes in ferroptosis, such as GPX4, TFRC, LOX, and ACSL4, are currently the research focus. ACSL4 catalyzes the conversion of CoA into specific polyunsaturated fatty acids. Extensive research indicates that the biosynthesis of arachidonoyl-CoA, facilitated by ACSL4, plays a pivotal role in the initiation of ferroptosis through the induction of phospholipid peroxidation. Hence, ACSL4 inhibition is the critical strategy to prevent ferroptosis. Evidence suggests that P-EVLP can regulate the interkingdom crosstalk between animals and plants by delivering effector molecules into human systems, thereby modulating cell signaling pathways [52]. In 2012, exogenous plant miR168a specifically targeted mammalian low density lipoprotein receptor adpator protein 1, which provided the first evidence of cross-kingdom regulation by miRNAs [53]. Later, these plant miRNAs were reported to be capsulated in EVs and delivered to mammalian cells. This phenomenon has been confirmed in several studies. For instance, cabbage-derived EVs achieved high encapsulation efficiency of miRNAs through transfection, with no observed changes in size (100 nm) or zeta potential (-14.2 mV) during the loading process [54]. In another study, miRNAs were successfully loaded into Acerola EVs by mixing them and subjecting the mixture to a heat shock after incubation on ice, with the highest efficiency observed after 30 min of incubation without further processing of the Acerola exosomes [55]. These studies demonstrate that plant miRNAs can be effectively encapsulated in EVs and potentially delivered to mammalian cells, providing scientific evidence for the potential role of plant miRNAs in interspecies communication. He et al. have identified 63 conserved and 30 novel miRNAs in H.cordata, among which miR159a is highly expressed, with TCF7 predicted as its target in humans [56]. However, miR159a is a widely present and highly conserved miRNA in plants that plays a role in plant growth, development, and environmental adaptation by targeting a class of regulatory genes known as GAMYB or GAMYB-like. Consistently, we also confirmed the high abundance of miR159a in HT-EVLP with the ability to target ACSL4, contributing to ferroptosis inhibition.

Several limitations should be considered in this study. While we explored the role of miR159a contained in HT-EVLP, other biochemical constituents may also contribute to its efficacy. After all, increasing evidence suggests that the functionality of P-EVLP is primarily due to the combined effects of its components. Apart from the BBB and neuron protection perspective, further studies are required to fully unravel the roles and protective mechanisms of HT-EVLP in cerebral I/R injury. Additionally, a thorough evaluation of the safety and pharmacokinetics of HT-EVLP is necessary before considering clinical application.

## Conclusions

This study verified that EVLP from H. cordata exerts potent neuroprotective effects and elucidated their broad protective mechanisms against cerebral I/R injury in both in vitro and in vivo models. HT-EVLP shows promise as a therapeutic agent or nucleic acid carrier for treating cerebral I/R injury.

## Data Availability

The data supporting this article have been included as part of the Supplementary Information.

## Abbreviations

BBB: Blood–brain barrier
CCA: Common carotid arter
DAPI: 4,6-Diamidino-2-phenylindole
ECA: External carotid artery
EVs: Extracellular vehicles
GSH: Glutathione
HT: Houttuynia cordata Thunb
HT-EVLP: Houttuynia cordata Thunb -derived EVs-like particles
I/R: Ischemia–reperfusion
ICA: Internal carotid artery
LDH: Lactate dehydrogenase
MCA: Middle cerebral artery
MCAO: Middle cerebral artery occlusion
NTA: Nanoparticle tracking analysis
OGD/R: Oxygen–glucose deprivation/reoxygenation
PBS: Phosphate-buffered saline
ROS: Reactive oxygen species
SOD: Superoxide dismutase
TEM: Transmission Electron Microscope
TTC: Tetrazolium dye
